# The MUC5B mucin polymer is dominated by repeating structural motifs and its topology is regulated by calcium and pH

**DOI:** 10.1038/s41598-019-53768-0

**Published:** 2019-11-22

**Authors:** Gareth W. Hughes, Caroline Ridley, Richard Collins, Alan Roseman, Robert Ford, David J. Thornton

**Affiliations:** 1 Wellcome Trust Centre for Cell-Matrix Research, University of Manchester, Manchester Academic Health Sciences Centre, Manchester, M13 9PT UK; 2 Lydia Becker Institute for Immunology and Inflammation, University of Manchester, Manchester Academic Health Sciences Centre, Manchester, M13 9PT UK; 3 School of Biological Sciences, Faculty of Biology, Medicine and Health, University of Manchester, Manchester Academic Health Sciences Centre, Manchester, M13 9PT UK

**Keywords:** Glycobiology, Electron microscopy

## Abstract

The polymeric mucin MUC5B provides the structural and functional framework of respiratory mucus, conferring both viscoelastic and antimicrobial properties onto this vital protective barrier. Whilst it is established that MUC5B forms disulfide-linked linear polymers, how this relates to their packaging in secretory granules, and their molecular form in mucus remain to be fully elucidated. Moreover, the role of the central heavily O-glycosylated mucin domains in MUC5B conformation is incompletely described. Here we have completed a detailed structural analysis on native MUC5B polymers purified from saliva and subsequently investigated how MUC5B conformation is affected by changes in calcium concentration and pH, factors important for mucin intragranular packaging and post-secretory expansion. The results identify that MUC5B has a beaded structure repeating along the polymer axis and suggest that these repeating motifs arise from distinct glycosylation patterns. Moreover, we demonstrate that the conformation of these highly entangled linear polymers is sensitive to calcium concentration and changes in pH. In the presence of calcium (Ca^2+^, 10 mM) at pH 5.0, MUC5B adopted a compact conformation which was lost either upon removal of calcium with EGTA, or by increasing the pH to 7.4. These results suggest a pathway of mucin collapse to enable intracellular packaging and mechanisms driving mucin expansion following secretion. They also point to the importance of the tight control of calcium and pH during different stages of mucin biosynthesis and secretion, and in the generation of correct mucus barrier properties.

## Introduction

Mucus is a complex and dynamic hydrogel that lines the luminal surfaces of mucosal epithelial cells which functions as a barrier protecting against pathogens and other foreign insults. Mucus layers are highly adaptive and respond to a plethora of different insults experienced at epithelial surfaces. As such these barriers are formed from tissue specific secretions and are tailored to the local environment and the distinctive challenges faced at each mucosal surface^1^. In disease states such as cystic fibrosis (CF), increased mucus concentration results in the formation of a highly adhesive and obstructive mucus layer^2,3^. Additionaly, alterations in the ionic content of the airway surface liquid may also be a factor that contributes to abberant mucus properties by affecting post-secretory mucin expansion^4^. The macromolecular changes that occur within the mucus barrier during CF progression are still yet to be fully elucidated and require an in-depth understanding of mucin and mucus structure, and how it relates to function.

The polymeric gel forming mucins MUC5B and MUC5AC form the structural backbone of airway mucus barriers. Arranged in a network-like formation, these large glycoproteins provide the gel-like properties of the mucus layer^5^. Mucins form long thread-like structures due to the biophysical constraints imparted by O-linked glycan chains which decorate the central region of the protein. These glycan chains represent a major biologically active region of the protein, and can act to maintain epithelial sheet hydration, prevent infection, and provide nodes of attachment for other secreted molecules^1,6,7^.

MUC5B is essential for effective mucociliary clearance^8^, and is a linear polymer formed via C-terminal dimerization and N-terminal multimerization mediated by disulfide linkages^9,10^. Structural techniques such as electron microscopy (EM), atomic force microscopy (AFM), small-angle X-ray scattering (SAXS), and small-angle neutron scattering (SANS) have provided new insights into mucin poymer topology, and highlighted the highly extended conformation of the glycosylated mucin domains^11–13^. While EM and SAXS analyses of MUC5B N- and C-terminal domains have demonstrated their globular nature^9,14^. Furthermore, through the use of tip-enhanced Raman spectroscopy, Davies and co-workers have demonstrated that the globular domains found within the MUC5B polymer are dominated by β-sheet structures, whilst the glycosylated mucin domains remain disordered^15^.

Recent evidence has shown that post-secretion, submucosal gland-derived MUC5B exists as part of complex higher-order structures termed mucin-bundles^16^. These structures are believed to be formed of many interacting mucin polymers and represent a novel concept in mucin network organization^17,18^, however the mechanistic details underpinning their formation remain to be elucidated. Changes to the supramolecular topology of MUC5B are important in both post-secretory expansion and during intragranular packaging. A decreasing pH and an increase in calcium concentration are proposed to drive mucin condensation via the formation of N-terminal multimers^9,19^. This compaction is essential for ordered packaging of MUC5B into the confines of the secretory granule^20^, but can lead to mucoviscidosis in CF due to aberrant mucin unfolding post-secretion^21^. Despite these recent advances gained from studies using recombinant mucin proteins^9,14^, it remains unclear as to how full-length mucin polymers are affected by changes in Ca^2+^ concentration and pH. This knowledge will be important to gain a more complete understanding of mucin packaging/unpackaging.

Here, using transmission-EM (TEM) combined with a directed single-particle analysis, we performed a structural analysis of MUC5B overall architecture. This enabled us to determine distinct motifs and features within the MUC5B polymer, that until recently was believed to exist as a highly disordered and unstructured macromolecule. Furthermore, it has recently been shown using recombinantly expressed proteins that Ca^2+^ plays a key role in modulating the formation of non-covalent assemblies of MUC5B N- and C-terminal domains^9,14^. However how calcium affects the structure of the MUC5B polymer remains unclear. We therefore performed structural comparisons of MUC5B polymers either in the presence or absence of calcium, which identified dramatic global changes in MUC5B topology, thus demonstrating that calcium is a key mediator of mucin network organization.

## Results

### Folded protein domains confer structural flexibility onto MUC5B, resulting in the formation of a highly flexible macromolecule

**Figure 1:**
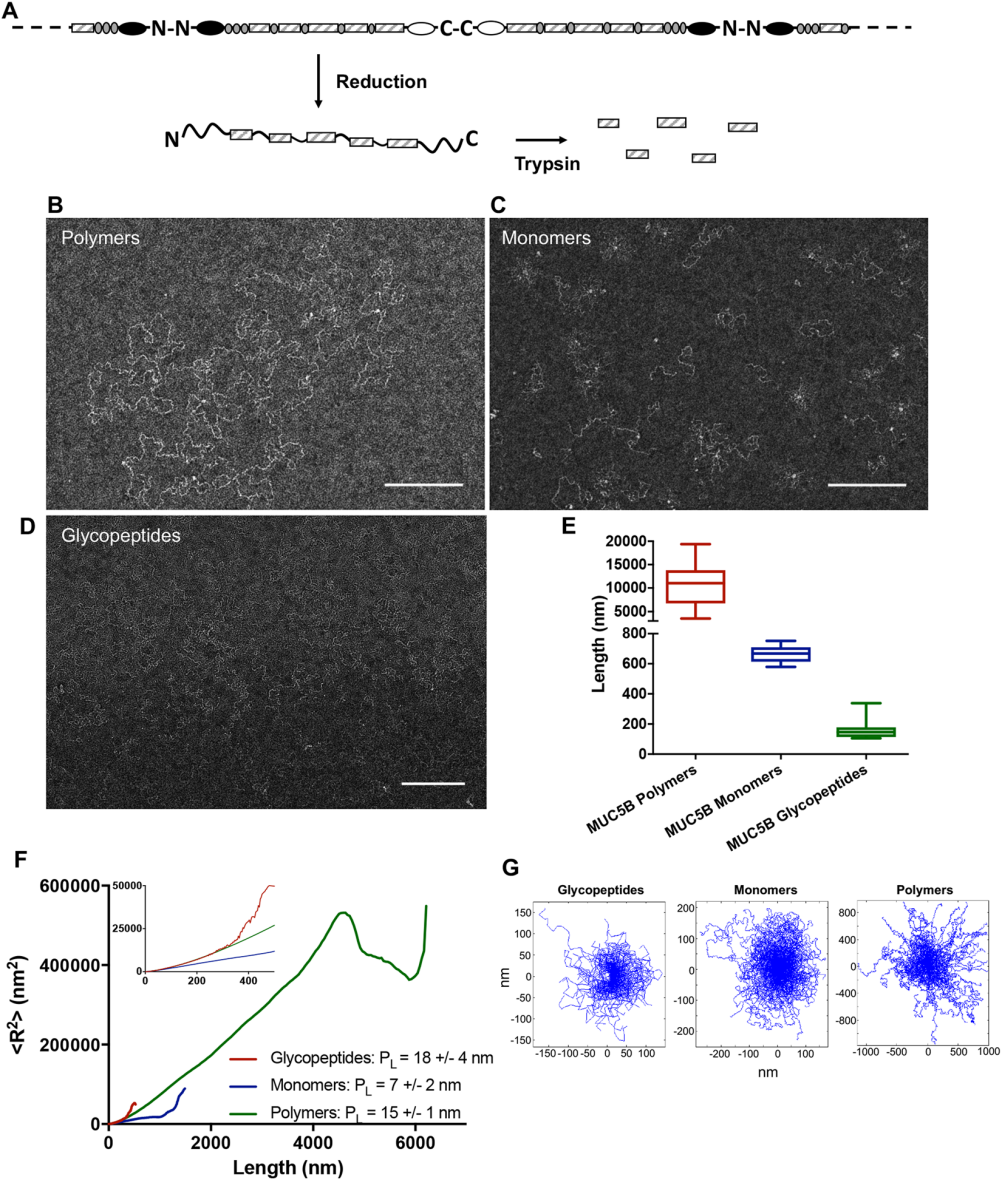
MUC5B displays considerable structural flexibility. (**A**) Cartoon highlighting the structural components of the MUC5B macromolecule. (**B**) Natively purified MUC5B polymers at 5 μg/ml were visualized through TEM. (**C**) MUC5B at 5 μg/ml was reduced and carboxymethylated to produce MUC5B monomers, which were subsequently visualized through TEM. (**D**) MUC5B at 5 μg/ml was reduced, carboxymethylated and trypsin treated to produce MUC5B mucin domain ‘glycopeptides’. The structures of which were imaged via TEM. Representative images are shown in B-D and the scale bars are 200 nm. (**E**) Following TEM visualization the contour lengths of MUC5B polymers, monomers, and glycopeptides were measured. (**F**) The persistence length of MUC5B and its constituent parts was determined by measuring the mean square of the end-to-end distance (R) as a function of *ℓ* (the distance between two distinct points along the chain contour). (**G**) MUC5B contour length traces. The initial tangents of each molecule are aligned at 0 nm to allow for the visualization of the different structures.

It is well established that MUC5B forms high molecular weight, linear polymers^22,23^, however, the architectural features of MUC5B remain ambiguous. To evaluate its supramolecular topology, we visualized MUC5B in polymeric, monomeric (reduced and carboxymethylated) and glycopeptide (reduced and carboxymethylated and then trypsin-treated) forms by using TEM (Fig. 1A). In the process of negative staining a polymer is embedded in a layer of heavy metal ions, which interact strongly with the electron beam. The stain exclusion pattern formed (hence negative staining) allows us to visualize overall organization of the molecule in a way that cryo imaging would not since MUC5B is a thin glycosylated polypeptide and this type of macromolecule has notoriously poor contrast.

MUC5B polymers, monomers and glycopeptides displayed highly flexible and entangled structures, appearing as linear macromolecules. As expected MUC5B displayed marked heterogeneity in terms of its size, polymers averaging 10.7 ± 1.6 μm in length (ranging from 4–20 μm), monomers averaging 671 ± 11 nm in length (ranging between 579–752 nm), and glycopeptides averaging 130 ± 6 nm in length (ranging between 105–308 nm) (Fig. 1B–E).

The dramatic structural transition that mucins experience post-secretion requires polymers to behave as highly flexible molecules^12^. This inherent flexibility allows mucins to structurally adapt to changes in the environment, an important factor in the formation of a functional mucus barrier. Recently, Georgiades and colleagues demonstrated the structural flexibility within MUC5AC and MUC2 polymers and reported mucin persistence lengths between 8–10 nm^13^. Whilst this illustrates the high levels of flexibility within the mucin polymer, it remains unclear as to how individual sub-domains contribute to this structural flexibility. Using TEM we measured the persistence lengths of MUC5B polymers, monomers, and glycopeptides in an attempt to define how flexibility is conferred onto MUC5B. Persistence length defines the stiffness of a polymer: molecules are considered to be flexible if the persistence length is considerably smaller than that of the polymer contour length, with shorter persistence lengths representing more flexible polymers^24^. Reduced monomeric MUC5B was found to be the most flexible molecule, with a persistence length of 7 ± 2 nm. Interestingly, MUC5B glycopeptides displayed the highest persistence length (18 ± 4 nm), with polymeric MUC5B presenting a persistence length of 15 ± 1 nm, thus suggesting that glycosylated mucin domains (studied here as glycopeptides) represent the least flexible region of the MUC5B molecule (Fig. 1F–G). The lower persistence length measured for monomeric MUC5B compared to MUC5B polymers may be explained through the reduction process, which resulted in unfolding of the globular protein domains (N- and C-terminal domains and internal cysteine-rich domains (Fig. 1A)) and increased flexibility.

While EM has been used as a method for determination of persistence length of biopolymers there are potential limitations with using this technique^25,26^. For example, the 2D representation of dynamic 3D molecules may be affected by differential interaction of the various forms of MUC5B (polymers, reduced monomers and glycopeptides) with the EM grid, for instance the higher proportion of proteinaceous regions in the polymers and reduced monomers may allow for enhanced adhesion to the surface. However, attachment to the grid is likely to be comparable for the 3 species analysed as they all have the major glycosylated domains and they all appear extended in a way that corresponds to their macromolecular properties (i.e. flexible thread-like molecules with different lengths (Fig. 1B–E)). The results also show consistent behaviour, which suggests a reproducible pattern of binding to the grid. Moreover, the persistence length measurements for the MUC5B polymer are comparable to those reported in other studies investigating either MUC5AC or MUC2 mucin polymers through either AFM or SANS, thus highlighting the inherent flexibility of polymeric mucins^12,13^.

Together these results suggest that the proteinaceous globular domains within MUC5B are responsible for conferring flexibility onto the polymer, with glycan-rich mucin domains forming considerably stiffer regions.

### MUC5B mucin domains contain distinct structural motifs

Through recent single particle EM and Raman spectroscopy analyses a more detailed map of MUC5B topology is emerging, in which highly ordered, globular N- and C-terminal domains are punctuated by mucin domains consisting of an unordered protein backbone in a random coil conformation^9,15^. The mucin domains act as the sites of heavy O-glycosylation, and it remains unclear as to whether this can confer some structure onto the polymer. To investigate domain structure within a full-length mucin, single particle EM analysis was performed and from this we were able to map structural motifs along the MUC5B polymer.

**Figure 2:**
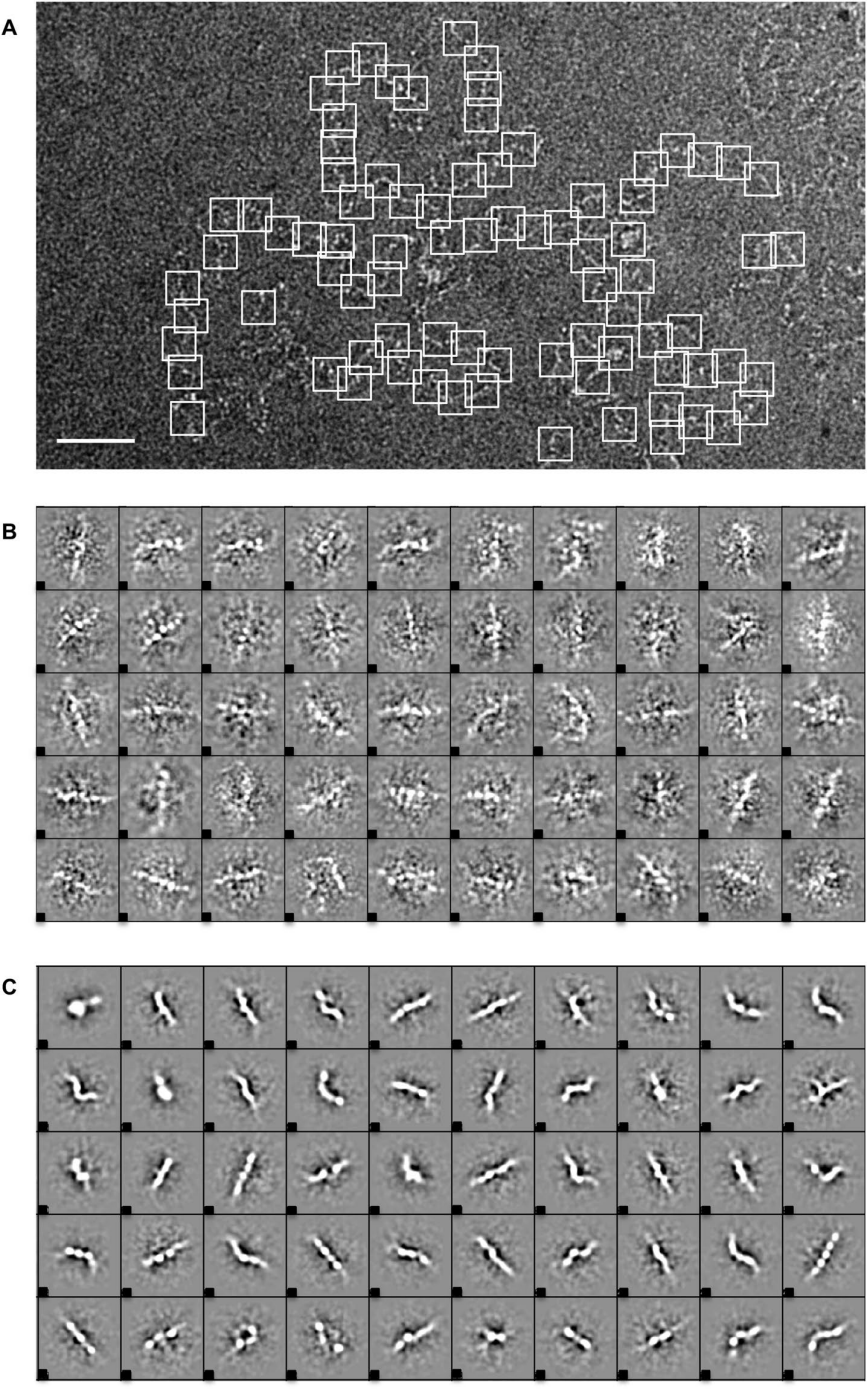
MUC5B contains repeating structural motifs. (**A**) MUC5B polymers were imaged using TEM. Regions along the polymer axis on the images were selected and isolated, using a 45 × 45 nm box, generating a set of ~9000 particles, which was subjected to iterative rounds of alignment and class averaging (using EMAN2^44^). Scale bar: 100 nm. Representative particles of which are shown in (**B**). (**C**) Fifty class averages were generated to highlight the distinct motifs found repeating throughout the polymer.

To structurally define the motifs present within the mucin polymer, MUC5B was negatively stained and imaged via TEM, with ~200 micrographs collected. Through the use of EMAN2.1 software, overlapping 45 × 45 nm boxes were used to pick particles covering the full length of the MUC5B polymer (Fig. 2A). Boxes were selected with minimal overlap and each area was manually selected so that a straight fiber region was central in each box; areas that were considerably bent or poorly stained were not selected^27^. Fifty class reference free class averages were generated from ~9000 particles after analysis following 10 rounds of iteration (Fig. 2B,C). Distinct structural motifs were observed from these class averages, representing both the globular and glycosylated domains within MUC5B. Classes consisting of a beaded topology were most commonly identified; these areas are apparent in the raw data but are more clearly defined following averaging. These beads of around 5 nm in diameter contained a distinct periodicity, and were found to repeat throughout the polymer.

**Figure 3:**
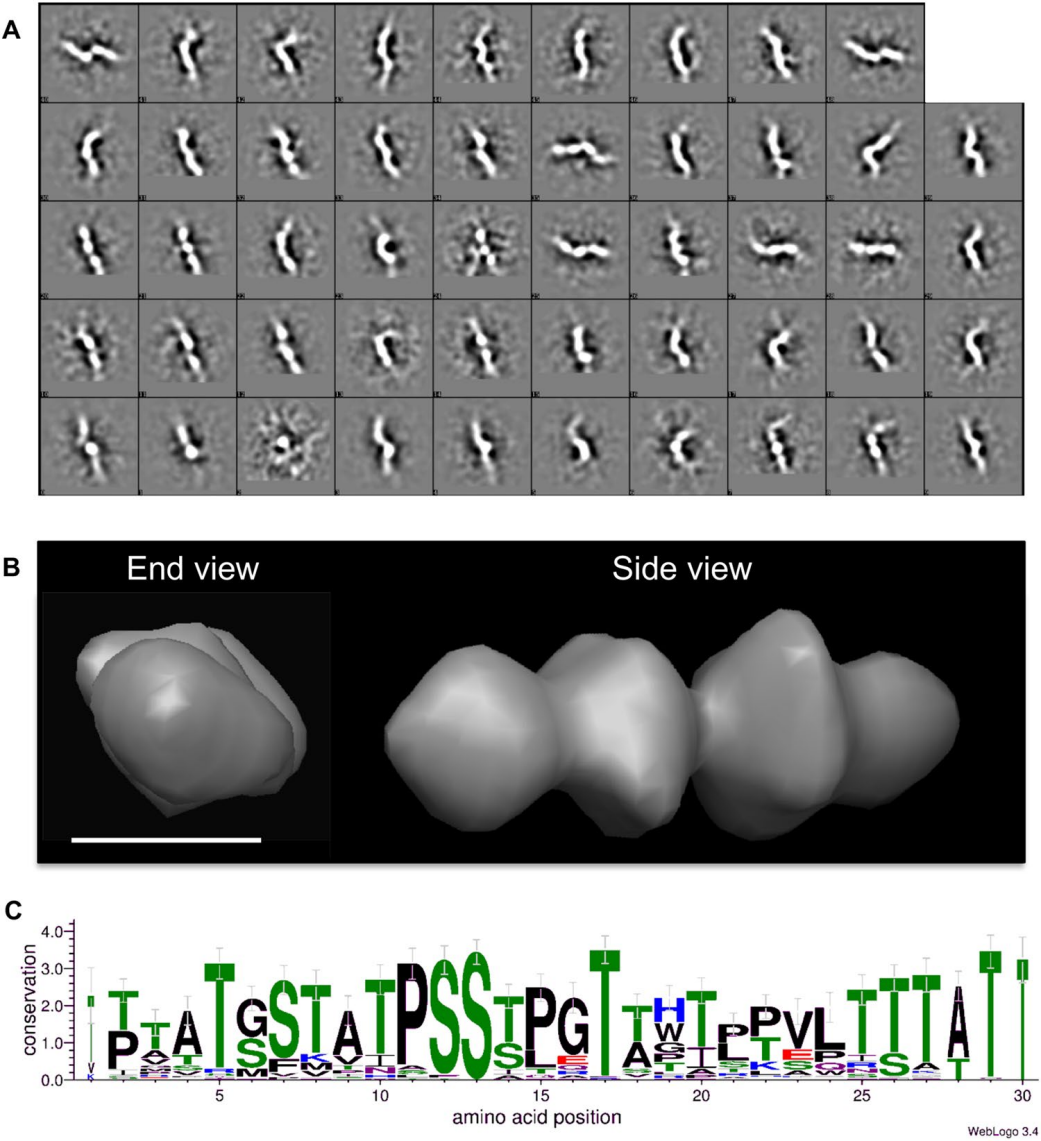
MUC5B glycopeptides form repeating beaded structures. (**A**) MUC5B glycopeptides were visualized through TEM, with single particles from individual glycopeptides molecules being boxed out (45 × 45 nm boxes) and subjected to iterative rounds of alignment and class averaging. (**B**) 3-D reconstruction of the MUC5B mucin domain. Particles were extracted from aligned glycopeptide fibers before being reconstructed into a 3D model using single particle reconstruction methods (using FindEM and SPIDER^39,40^). Scale bar: 5 nm. (**C**) Sequence logo highlighting the conservation of amino acids within MUC5B consensus repeats. These repeats are dominated by both serine and threonine residues (shown in green), which represent the sites of the O-linked glycans that characterize mucin domains. Punctuating these glycosylation sites are predominantly hydrophobic amino acids (shown in black/blue/purple).

To define where these structural motifs are from within the MUC5B polymer, we subjected MUC5B to reduction and alkylation followed by trypsin digestion, thus isolating the glycosylated mucin domains away from the ‘naked’ globular regions of the polymer, which are destroyed by this treatment. These glycopeptides were then negatively stained and imaged via TEM. Again using a 45 × 45 nm box and the same strategy ~6000 particles were picked, thus covering the full length of the MUC5B mucin domains. Fifty class averages were generated from these particles (Fig. 3A). A beaded motif, ranging between 4–7 nm, was observed in the majority of classes. A 3-D model of the MUC5B glycopeptides was subsequently generated using SPIDER software. MUC5B mucin domains were seen to consist of a repeating beaded topology, with ‘beads’ forming globular structures of around 5 nm in diameter (Fig. 3B).

Recent structural investigations have demonstrated that MUC5B mucin domains exist in an unordered conformation. Despite this disorder, structural patterns derived from regions of glycosylation are believed to confer some structure onto the mucin backbone^15^. It is feasible that the beaded topology we present here represents these glycosylation patterns along the mucin domain. To investigate this we performed sequence analysis studies on the MUC5B mucin domain. Using RADAR software we identified 72 repeating sequences present in the central glycosylated domains of MUC5B. These 72 repeat sequences were then aligned and analyzed using the WebLogo 3.4 software to create a sequence logo representing the MUC5B consensus sequence (Fig. 3C). The logo consists of a 30 amino acid sequence that is repeated throughout the MUC5B mucin domains, and provides us with detailed information about amino acid conservation within these regions. A recent study into intrinsically disordered peptides from the O-glycan attachment region of aggrecan (a chondroitin sulfate-containing proteoglycan) has suggested that when unglycosylated these molecules contain semi-ordered, stiffened structures with a mass per unit length of 770 Da nm^−1^ that was reduced by 25% after glycosylation (~620 Da nm^−1^). Assuming the average mass of amino acid to be 100 Da this would equate to ~6 amino acids per nm^28^. Thus it is it is attractive to hypothesize that the 5 nm bead structures reported here represent a single 30 amino acid repeat, perhaps with the non-glycosylated PPVL sequence forming a linker region between beads.

Together these results provide new structural insights into MUC5B topology, and show that distinct structural motifs are found within glycosylated mucin domains, which were previously thought to consist of unordered random coils.

### Calcium is a key regulator of MUC5B network topology

Calcium has recently been shown to mediate the formation of homotypic MUC5B N-terminal domain interactions, and heterotypic N- and C-terminal domain interactions which are thought to drive the changes in conformation necessary for compaction into the granule^9,14,19^. However, the use of recombinantly expressed domains in these studies leaves it unclear as to how calcium can structurally affect full-length polymeric MUC5B. To investigate how calcium and pH can influence mucin structure, we visualized MUC5B via TEM at varying pH and calcium concentrations and assessed changes in macromolecular conformation.

**Figure 4:**
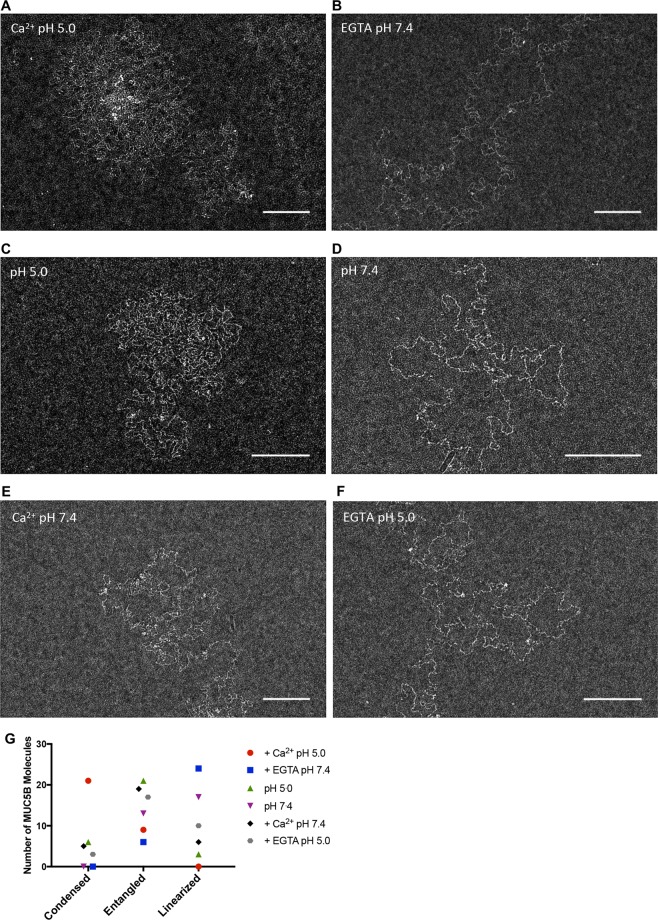
Calcium promotes MUC5B condensation. (**A**–**F**) Natively purified MUC5B at 5 μg/ml was visualized by TEM untreated at either pH 7.4 or pH 5.0 or after incubation with either 10 mM Ca^2+^ or 10 mM EGTA. (**G**) Visualised molecules (30 from each treatment) were classified into 3 categories condensed, extended or linearized that best described their appearance.

MUC5B was subjected to a low pH, high calcium environment (pH 5.0, 10 mM Ca_2_^+^). Under these conditions, MUC5B formed highly condensed structures, polymers appeared as compact units, with individual chains indistinguishable from one another. In many cases the central region of these structures contained dense material, consisting of nodes around which the polymer chains were organized (Fig. 4A). These structures were dramatically reversed upon calcium chelation via treatment with 10 mM EGTA at pH 7.4. In the absence of calcium MUC5B appeared as linearized, individual fibers, resulting in an open and expanded mucin network (Fig. 4B).

To further assess the role calcium plays in defining mucin structure, we varied pH levels without changing the calcium concentration of the MUC5B sample. At pH 5.0 mucins formed condensed structures (Fig. 4C); these molecules however did not form the unique structures seen in the presence of 10 mM calcium at pH 5.0, with the central node region absent from these structures. In contrast, at pH 7.4 MUC5B appeared as a linear network of polymers, however the straightened appearance of MUC5B after treatment with 10 mM EGTA at pH 7.4 was not observed, these molecules adopting a more entangled conformation (Fig. 4D).

To dissect the independent effects of calcium and pH on MUC5B structure, we imaged MUC5B in the presence of 10 mM calcium at pH 7.4 and EGTA at pH5.0. MUC5B formed similar structures under both of these conditions, however polymers were seen to be slightly more compact in the presence of calcium pH 7.4 (Fig. 4E), compared to EGTA pH 5.0 treated molecules (Fig. 4F), suggesting both calcium and pH can affect mucin conformation, but together have a major synergistic effect on macromolecular structure.

Kesimer and collegues have previously utilized electron microscopy techniques to visualize MUC5B at different stages of maturation after secretion, allowing for a classification of these molecules based on conformational differences^20^. Here we have used a similar classification method to categorize MUC5B polymers as condensed (e.g. Figure 4A,C), entangled (e.g. Figure 4D–F) or linearized (e.g. Figure 4B), thus allowing quantification of the structural differences highlighted above (Fig. 4G).

**Figure 5:**
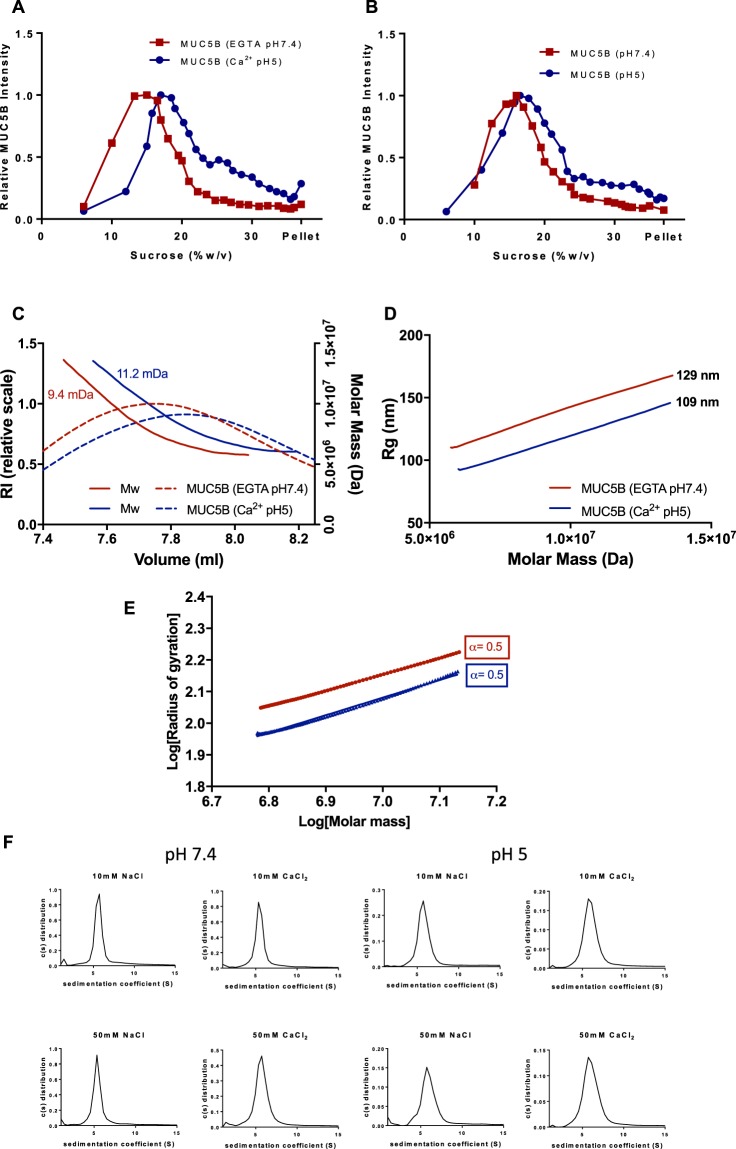
MUC5B polymers are condensed in the presence of calcium at pH 5.0. The effects of changes in calcium concentration and pH on mucin behavior were assessed through sucrose density rate zonal centrifugation and SEC-MALLS analyses. (**A**) MUC5B at 50 μg/ml was incubated with either 10 mM Ca^2+^ at pH 5.0, or 10 mM EGTA at pH 7.4. Changes in the MUC5B sedimentation profile were assessed by immunodetection with the MAN-5BIII antiserum. (**B**) 50 μg/ml purified MUC5B at either pH 7.4 or pH 5.0 was subjected to rate zonal centrifugation, sedimentation behavior was assessed by immunodetection with MAN-5BIII. (**C**) 50 μg/ml MUC5B incubated with either 10 mM Ca^2+^ at pH 5.0, or 10 mM EGTA at pH 7.4 was analyzed by SEC-MALLS. Calcium treatment was seen to increase the molecular weight of MUC5B as compared to EGTA treated polymers. The dashed lines show the refractive index (RI) profiles. (**D**) The average MUC5B radius of gyration was decreased in the presence of calcium at pH 5.0, as compared to EGTA treated molecules. (**E**) The relationship between size and shape for molecules can be expressed by the Mark-Houwink parameter α, obtained from the relationship R_G_ = AMr^α^, where A is a constant relating to the mass per unit length of the molecule. α can be used as an index of the stiffness of MUC5B; α = 1 for rod-like molecules, 0.5–0.6 for random coils and 0.33 for compact spheres. The plot shows that under both conditions MUC5B behaves as a coil in solution. (**F**) 1 mg/ml MUC5B glycopeptides incubated with NaCl (10 mM and 50 mM) or CaCl_2_ (10 mM and 50 mM) at pH 7.4 or pH 5.0 were subjected to AUC. The sedimentation coefficient for these molecules remained constant under all conditions.

Owing to the augmented effects that calcium and pH has on MUC5B structure we decided to focus on how calcium and low pH together can influence the biophysical properties of the MUC5B polymer. Changes to the in solution behavior of MUC5B in response to altering calcium concentration and pH were determined through sedimentation analysis using rate zonal centrifugation and SEC-MALLS analyses. In the absence of calcium (EGTA treatment) at pH 7.4, MUC5B sedimented slowly, forming a peak at ~15% sucrose. In contrast, addition of excess calcium and reduction in pH to 5.0 resulted in a shift in mucin sedimentation profile, with MUC5B sedimenting faster and over a broad range of sucrose concentrations (18–30%) (Fig. 5A). Furthermore, it was observed that pH alone can affect mucin sedimentation behavior. Reduction in pH from 7.4 to 5.0 resulted in a moderate shift in the sedimentation profiles of MUC5B, with faster sedimenting molecules detected at lower pH (Fig. 5B). This shift, however, was less pronounced than with 10 mM Ca^2+^ pH5 treated samples. In addition, SEC-MALLS revealed an increase in apparent MUC5B molecular weight from 9.6 mDa in EGTA pH 7.4 treated samples, to 11.2 mDa upon calcium treatment at pH 5.0 (Fig. 5C). This apparent increase in molecular weight is indicative of calcium driven transient crosslinks within the MUC5B network. Furthermore, calcium treatment at pH 5.0 resulted in a reduction in the radius of gyration of MUC5B, as compared to EGTA treated molecules, from 129 nm to 109 nm (Fig. 5D), indicating the ability of calcium to drive mucin condensation. Through the determination of the Mark-Houwink parameter, α (which acts as a measure of molecular stiffness;^29^), we observed no coil to globule transition, with MUC5B polymers remaining as coils (α = 0.5) in the both the presence and absence of calcium (Fig. 5E). As such the effect of calcium on MUC5B represents a transition from a stiffened random coil of a linear MUC5B polymer to a cross linked more compact form of the polymer.

We next performed analytical ultracentrifugation to assess if the highly glycosylated mucin domains contributed to the calcium- and pH-dependent changes in MUC5B topology described above. The sedimentation coefficients of MUC5B glycopeptides (1 mg/ml) were found to be unaffected in both the presence (up to 50 mM) and absence of calcium, regardless of acidic (pH 5.0) or neutral (pH 7.4) pH (Fig. 5F).

Together these results demonstrate the importance of calcium and pH in controlling MUC5B polymer structural topology, previously only proposed by studies on recombinant MUC5B N- and C-terminal protein domains^9,14^. Here, direct assessment of MUC5B polymers provides evidence extending our knowledge of mucin structural organization by showing that MUC5B polymers adopt highly organized and condensed structures, driven by calcium and pH-dependent interactions between proteinaceous domains. Thereby providing further insight into how full length MUC5B polymer structure could be altered in conditions where there is an altered extracellular milieu such as in CF.

## Discussion

**Figure 6:**
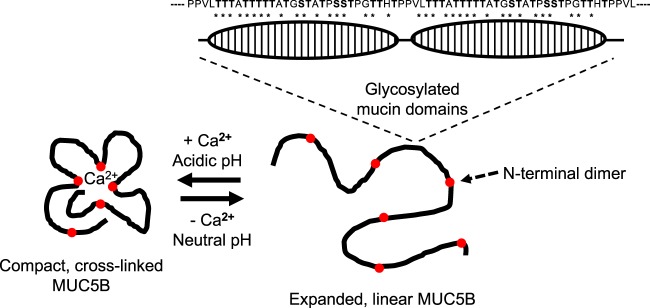
Model of MUC5B structural organization and how this is affected by calcium ions. Under the conditions prevailing in the mucin secretory granule (acidic pH and high calcium concentration) MUC5B condensation occurs through the formation of non-covalent, calcium-dependent homotypic interactions between mucin N-terminal dimers and heterotypic interactions beween N-terminal dimers and C-terminal dimers (the heavily glycosylated mucin domains having no direct role in this structure), which results in a highly organized and compact polymer^9,14,37^. The role of the internal Cys-rich domains of MUC5B in this process have yet to be investigated. Chelation of calcium and increase in pH (the environment following secretion into the extracellular mucus layer) results in mucin linearization and structural remodeling. Airway dehydration and ineffective calcium chelation, as proposed in CF^33,45^, will result in defective expansion and altered mucus properties. The overall structural topology of the MUC5B polymer is intrinsically linked to its glycosylation state, glycan patches (hatched ovals) impart structural organization onto the MUC5B polymer. *Denote potential O-glycosylation sites.

Here, we present a novel demonstration of the use of single particle analysis on full length MUC5B, allowing us to define distinct gross level structural motifs throughout the polymer. Through single particle analysis of isolated MUC5B mucin domains we have been able to identify structural motifs present in the highly glycosylated regions of the polymer, that likely represent gross repeats of the protein and the associated but realtively disordered glycosylation. Glycopeptide class averages show that mucin domains, despite heavy glycosylation, contain distinct units with a unique structure. These motifs manifest as ‘bead-like’ structures, which repeat along the polymer axis (Fig. 6), the diameter of which varied between 4–5 nm, which is in line with work from Round and colleagues who through AFM measurents reported the diameter of MUC5AC at ~3 nm^12^. Whilst no direct structural comparisons between MUC5B and MUC5AC have been performed, we suggest the comparable diameter measurements likely reflects similar levels of glycosylation present on both macromolecules. This work supports that performed by Davies and colleagues who alluded to the possibility of glycosylation patterns decorating the mucin domain^15^. In light of this we suggest that individual patches of glycosylation can confer order and structure onto these regions. In support of this, recent studies into intrinsically disordered polypeptides have suggested that these sequences do in fact contain semi-ordered structures, which form stiffened conformations when glycosylated, thus providing evidence for how mucin domains may be organized^28^. This view of the mucin domain as an organized molecular region, containing independent patches of structured glycans represents a novel concept, one which adds detail to the idea that mucin domains adopt a conformation that resembles a bottlebrush^30^. Through sequence analysis of the MUC5B mucin domain we have mapped these patches of glycosylation to distinct repeating amino acid sequences; we therefore suggest that the beaded structure presented here is a consequence of the ordered glycosylation of these repeating sequences (Fig. 6). Considering these repetitive 5 nm beads in the context of the the 18 nm persistence length measured for MUC5B glycopeptides allows us to draw conclusions about the inherent flexibility of the mucin molecule. The observed 5 nm substructure implies that an intrinsic level of structural organization exists within the MUC5B glycopeptide but with flexible, end to end contacts between beads that can be bent over a 18 nm scale.

Despite recent advances in understanding how mucins are structurally organized^7,9^, there remains a lack of knowledge regarding how mucins structurally form a functional mucus barrier, and how this structure is modified in conditions characterized by changes in mucus ionic content. Collectively, the data presented here highlights the inherent structural flexibility of MUC5B and provides us with new insights into how changes to mucus ionic content can alter mucin conformation. It is well established that in disease states such as CF, mucus barrier architecture is compromised. Through mimicking the elevated calcium environment (2–4 mM) found within the CF mucus barrier^31,32^, we provide structural information about how polymeric MUC5B may be structurally arranged in CF. Furthermore, owing to the acidic pH (~pH 6) and high calcium environment of the secretory granule (~200 mM), the structures we present here may also relate to some apsects of the intracellularly packaged mucins^33^. MUC5B displayed dramatic structural sensitivity in response to calcium treatment at pH 5.0. The highly condensed structures showed a reduction in radius of gyration and a concurrent increase in density. These compact molecules displayed remarkable similarities to the modelled structures proposed by^9^ in which proteinaceous nodes consisting of N-terminal domain dimers are proposed to interact to form a focal point around which glycosylated mucin domains are organized. It is feasible therefore that these highly organized and compact structures denote an accurate representation of some aspects of packaged mucins. Similar MUC5B topology has been presented by Kesimer and colleagues who observed compact polymers organized around numerous nodes of 10–20 nm in diameter^20^. We suggest that only within a acidic pH, high calcium environment (as found within the secretory granule) are the organized structures dominated by electron dense cores created (Fig. 6).

The formation of a functional mucus barrier relies on mucin transition from a condensed form to an expanded, linear conformation. This macromolecular reorganization is thought to occur rapidly post-secretion^34^, with mucin expansion driven by a process of counter-ion replacement (Ca^2+^ ions replaced by Na^+^)^35^. The resulting change in the osmotic pressure of the mucus system is believed to trigger the phase transition from condensed to expanded states^36^. Increasing the calcium concentration of the extracellular milieu can dramatically hinder mucin expansion and mucus swelling^32^, thus highlighting the importance of maintaining a regulated ionic balance within the mucus barrier. Therefore, by removing calcium via EGTA chelation, and maintaining pH at 7.4 we aimed to mimic the ionic conditions found in the extracellular space. Our data shows that under these conditions MUC5B adopts an open and expanded conformation, with the mucin network consisting of highly linearized and straightened polymers, a topology which may represent a mature mucin network. This supports work from Kesimer and colleagues who postulated that the post-secretion change in ionic content was the driving factor behind mucin expansion^20^. In addition, we provide evidence to suggest that the calcium driven condensation of MUC5B occurs through interactions within the proteinaceous domains of MUC5B alone, with calcium having little influence on interactions between mucin domains. These results when taken together with work from Raynal and colleagues, who demonstrated that calcium-dependent mucin crosslinking is lost upon reduction, or through treatment with chaotropic agents^37^, provides us with a more robust understanding of mucin compaction, and one which emphasizes the importance of the non-glycosylated domains within MUC5B as key regulatory components of the polymer.

The ability of natively purified mucins to transition from condensed to expanded states provides us with important information about mucin structural flexibility and post-secretion processing. The ability of MUC5B to readily alter conformation in response to changes in ionic content, adopting potentially both intragranular and extracellular structures would suggest these molecules remain intact in an adaptable yet stable form. This is in line with work from Ridley and colleagues who showed that MUC5B N-terminal domains remain intact following granule release^10^. Furthermore, through comparing the relationship between log Rg and log average molecular weight we have shown that the ability of MUC5B to produce rapid changes in conformation through calcium driven condensation is indicative of network crosslinking rather than a coil to globule transition.

Here we establish a relationship between changes in ionic environment and structural changes to the MUC5B network. Furthermore, we show that mucin polymers are inherently flexible and that the calcium driven changes to MUC5B conformation are reversible. This structural plasticity is of major importance when considering potential treatments for CF lung disease: treatments must be able to structurally influence the mucin network and reverse mucin condensation. The displacement of calcium from within the CF mucus barrier is considered an important factor in driving this conformational change^32^, and we have visualized how MUC5B responds to treatment with the calcium chelator EGTA. The observed MUC5B linearization in response to the removal of calcium provides us with information about how mucins are organized within a healthy mucus barrier and allows us draw conclusions about how potential CF treatments must structurally affect the topology of the mucin network.

## Experimental Procedures

### Native MUC5B purification

Whole human saliva donated from 10 healthy individuals was pooled and solubilized in 0.1 M NaCl, 20 mM Tris pH 7.4, allowing for native MUC5B polymers to be purified via an adapted two-step isopycnic density gradient centrifugation process as previously described^37^. Briefly, following saliva solubilization, CsCl was added to the sample producing a starting density of 1.4 g/ml and subjected to density-gradient centrifugation for 65 hours at 40000 rpm and 15 °C using a Beckman OptimaTM L-90K Ultracentrifuge in a Beckman Ti45 rotor. After centrifugation, mucin-containing fractions were pooled and subjected to a second round of isopycnic centrifugation in CsCl at a starting density of 1.5 g/ml. Samples were centrifuged under the previous conditions and mucin-enriched fractions were then pooled and subjected to buffer exchange into 10 mM NaCl/10 mM Tris-HCl, pH 7.4.

Ethical approval for this research was acquired from the University of Manchester Research Ethics Committee (reference number 08293) and informed consent was obtained from all participants. All research was performed in accordance with the relevant guidelines and regulations.

### Mucin monomer and glycopeptide preparation

Mucin monomers were generated through a process of reduction and carboxymethylation. Purified mucins were reduced via incubation with 20 mM dithiothreitol (DTT) for 3 hours at 37 °C. Samples were then alkylated with 50 mM iodoacetamide (IAA) in the dark at room temperature for 45 minutes. DTT and IAA were removed from the mucin monomers by buffer exchange into 10 mM NaCl/10 mM Tris-HCl, pH 7.4 using a Vivaspin 5–10 kDa MWCO column.

Mucin domains are heavily glycosylated and resistant to tryptic digestion. To generate these large glycopeptides, mucin monomers were prepared as described above. 0.1 M ammonium bicarbonate was subsequently added to the sample at a 1:1 ratio. Proteomic grade trypsin (Promega, Madison, USA) was then added to the sample at a 1:20 (w/w) ratio and incubated overnight at 37 °C. Mucin glycopeptides were recovered from the digested sample using a Vivaspin 5–10 kDa MWCO column, which after centrifugation retained the glycosylated mucin domains, and allowed through the digested tryptic peptides. The mucin glycopeptides were subsequently exchanged into 10 mM NaCl/10 mM Tris-HCl, pH 7.4 using a Vivaspin 5–10 kDa MWCO column.

To confirm the generation of MUC5B monomers and glycopeptides these molecules were subjected to SEC-MALLS analysis and compared to polymeric MUC5B samples (Supplementary Fig. 1).

### MUC5B treatments

The conformation of MUC5B is sensitive to ionic changes in the mucus barrier, to investigate this sensitivity, purified MUC5B was subjected to varying conditions and assessed for changes in structure. Purified MUC5B samples were diluted in 10 mM NaCl, 10 mM Tris pH 7.4 producing varying concentrations between 0.5 μg/ml and 1 mg/ml. Mucins were then pH adjusted to pH 5.0 or 7.4, and incubated for 24 hours at 4 °C with either 10 mM CaCl_2_ or 10 mM EGTA.

### Immunodetection

Samples (50 μl) were slot blotted onto the nitrocellulose membrane using a Minifold II 72 well slot blot manifold. Following slot-blotting nitrocellulose membranes were briefly rinsed in ddH_2_O and then blocked for 1 hour with 5% (w/v) dried milk powder dissolved in PBS pH 7.4. Membranes were then washed for 1 minute in 10 mM Tris-base/150 mM NaCl/0.1% (v/v)/Tween-20, pH 8.0 (TBST) before being incubated for 12 hours with MAN-5BIII, rabbit polyclonal (1:2000 dilution) antiserum^38^ dissolved in TBST. After 3 × 5 minute washes in TBST the membrane was subsequently incubated in the dark for 1 hour with IRDye 800CW Donkey Anti-Rabbit IgG (1:10000 dilution; Li-COR Biosciences, Lincoln, USA) secondary antibody. After this incubation the membrane was again washed 5 times for 5 minutes in TBST. Secondary antibodies were visualized and densitometry performed on band intensities using a LI- COR Odyssey® CLx Infrared Imaging System (Li-COR Biosciences, Lincoln, USA).

### Transmission electron microscopy

Samples were incubated for 30 seconds on carbon coated 400 mesh copper grids (Electron Microscopy Sciences, Pennsylvania, USA) that had been glow discharged at 30 Volts for 30 seconds. Grids were then washed in ddH_2_O for 10 seconds before negative staining with 2% (w/v) uranyl acetate (Agar Scientific, Essex, UK) for 1 minute. TEM data were recorded using a Tecnai BioTwin microscope at 100 Kv, and micrographs acquired using a Gatan Orius CCD camera. The nominal magnification was 23000 times, giving a sampling of 3.5 Å/pixel. Images were recorded between 0.5 and 5.0 nm defocus.

### Single particle analysis

Particles were manually picked along the mucin chain into 128 pixel boxes. Datasets initially contained ~9000 unique particles and prior to class averaging were subjected to contrast transfer function (CTF) correction, and filtered using a 20 Å low-pass Gaussian mask; since negative stain has a resolution of 2–3 nm higher resolution information is simply noise and reduces contrast. Particles then underwent 12 iterative rounds of reference-free class multi statistical class averaging using either cross-correlation scoring or the Fourier ring correlation as the main aligner, generating 2-D classes. No restrictions were placed on translational or rotational alignments. Classes that represented the same domain and had the same beading repeat were identified and grouped together into individual datasets, from which they were subjected to a second round of iterative class averaging.

The generation of a 3-D model from MUC5B glycopeptide particles was performed as previously described^39^. Firstly, the 9000 particles from the MUC5B glycopeptide dataset were imported into the SPIDER software and an initial template image was generated, to avoid reference bias this initial template was the average image of the unaligned stack of particles. Particles were then rotationally and translationally aligned in an iterative fashion to the initial template using the local projection-matching program FindEM^40^. This was iterated over 15 rounds, with the template updated to be the new average of aligned particles after each round. Unaligned particles were subsequently discarded after every round of iteration. Using SPIDER software the average of the aligned images was back-projected applying C-100 symmetry (perpendicular to the fibre axis) to generate an initial 3-D model. Using a projection matching procedure through FindEM and SPIDER software the initial model was refined in an iterative fashion. The initial model was back projected at incremental angles of 5° around the fiber axis to give a sufficient angular sampling. Using FindEM the model projections were used as references for multi-reference based rotational and translational alignment. Through cross correlation of model projections with the particle dataset, particle orientation was assigned, particles with the same orientation were summed into class averages and back projected in SPIDER allowing for the construction of a new model. This procedure was repeated until a stable model was generated.

### Mucin measurements

Using ImageJ software, the segmented line tool was employed to measure different mucin parameters, including, chain length and chain width. Differences in these measurements were statistically analyzed through the use of parametric unpaired t-tests using GraphPad prism software.

Persistence length measurements were performed using the Easyworm software suite^24^. Using electron micrographs recorded at 23000x magnification the persistence length of ~300 individual MUC5B polymers, monomers, and glycopeptides were measured. Using the Easyworm fitting software the contour of the mucin chain was traced, allowing for the fitting of a parametric spline along the length of the molecule. From the mucin contour length and shape the Easyworm software was able to derive persistence length (P) by measuring the mean square of the end-to-end distance (R) as a function of *l* (the distance between two distinct points along the chain contour).

### Rate zonal centrifugation

Rate zonal centrifugation was performed on 12 ml linear 10–35% sucrose gradients in PBS at pH 7.4, as previously described^41^. 500 μl samples were layered on top of the gradient and centrifuged at 21000 g for 90 minutes at 15 °C in a Beckman L-90 ultracentrifuge, using a Beckman SW40 Ti swing-out rotor. After centrifugation, tubes were fractionated from the top of the gradient and 24 fractions collected. Pelleted material at the bottom of the tube was recovered through solubilization in 6 M urea (VWR, Leicestershire, UK). The sucrose concentration of each fraction was determined through measurement of refractive index. Fractions were then analyzed for mucin distribution via immunodetection.

### Size-exclusion chromatography-multi-angle laser light scattering (SEC- MALLS)

Samples were applied onto a Superose 6 10/30 column (GE Healthcare, Amersham, UK), and eluted in 0.2 M NaCl/0.01 M sodium azide at a flow rate of 0.31 ml/min. Eluents were passed through a Wyatt inline DAWN EOS laser photometer followed by an Optilab rEX refractometer with quasielastic light scattering dynamic light scattering attachment (Wyatt Technology, Suffolk, UK). Using a dn/dc value of 0.165 ml/g, light scattering and refractive index measurements were derived and analyzed using ASTRA 6 software (Wyatt, Santa Barbara, USA).

### Analytical ultracentrifugation

MUC5B mucin domain glycopeptides were generated as stated above, and adjusted to 1 mg/ml in 10 mM Tris (and either 10 mM or 50 mM NaCl or CaCl_2_ at varying pH). Samples were analyzed using velocity experiments on an Optima XL-A ultracentrifuge (Beckman Instruments) at 45,000 rpm, as previously described^9^. The sedimentation coefficients were determined using SedFit version 13.0b^42^.

### Generation of a sequence logo of the central glycan-rich region of MUC5B

The full-length sequence of MUC5B (Accession number: Q9HC84) was analyzed using RADAR software, which identified 72 repeating sequences present in the central mucin domains of MUC5B. These 72 repeat sequences were then aligned and analyzed using the WebLogo 3.4 software^43^ to create a sequence logo representing the MUC5B mucin-domain consensus sequence.

## Supplementary information

Supplementary Figure 1

## Data Availability

All data generated or analysed during this study are included in this article. The EM density map is available upon request.
