# Supplementary material for: The MUC5B mucin polymer is dominated by repeating structural motifs and its topology is regulated by calcium and pH
Source: Sci Rep. 2019 Nov 22;9:17350. doi: 10.1038/s41598-019-53768-0 (PMC6874590; doi:10.1038/s41598-019-53768-0)
Supplement: Supplementary file 1 — Supplementary Figure 1 [file 41598_2019_53768_MOESM1_ESM.pdf]

SUPPLEMENTARY INFORMATION

The MUC5B mucin polymer is dominated by repeating structural motifs and its topology is regulated by calcium and pH

Gareth W Hughes[1,3], Caroline Ridley[1,3], Richard Collins[3], Alan Roseman[3], Robert Ford[3] and David J Thornton[1,2,3]

[1]Wellcome Trust Centre for Cell-Matrix Research and [2]Lydia Becker Institute for Immunology and Inflammation, [3]School of Biological Sciences, Faculty of Biology, Medicine and Health, University of Manchester, Manchester Academic Health Sciences Centre, Manchester, M13 9PT

To whom correspondence should be addressed: David J Thornton, Faculty of Biology, Medicine and Health, AV Hill Building, University of Manchester, Oxford Road, Manchester, M13 9PT. Tel: +44 (0)161 275 5647; E-mail: dave.thornton@manchester.ac.uk

Running title: Regulation of MUC5B polymer structure

Keywords: MUC5B, mucin, mucus, electron microscopy, cystic fibrosis, calcium.

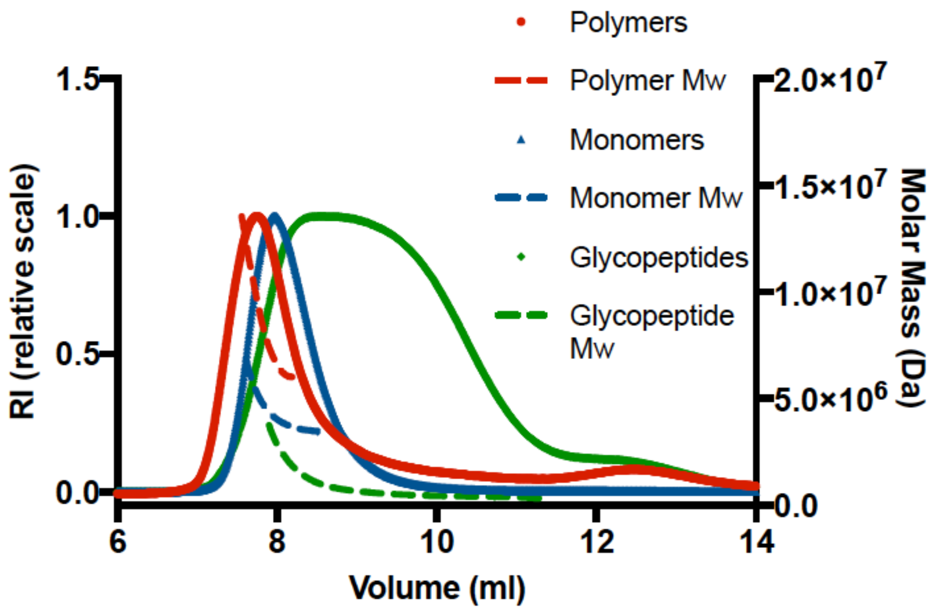

Supplementary Figure 1.

In order to determine differences in molecular weight (Mw) between polymeric (red), monomeric (blue) and glycopeptide (green) forms of natively purified salivary MUC5B molecules, SEC-MALLS was performed. Solid lines represent refractive index profiles, whilst dashed lines show molecular weight information. The average molecular weight of MUC5B polymers, monomers and glycopeptides was 12.5 MDa, 3.1 MDa 0.5 MDa, respectively.
